# Diverse Effects of an Acetylcholinesterase Inhibitor, Donepezil, on Hippocampal Neuronal Death after Pilocarpine-Induced Seizure

**DOI:** 10.3390/ijms18112311

**Published:** 2017-11-02

**Authors:** Jeong Hyun Jeong, Bo Young Choi, A Ra Kho, Song Hee Lee, Dae Ki Hong, Sang Hwon Lee, Sang Yup Lee, Hong Ki Song, Hui Chul Choi, Sang Won Suh

**Affiliations:** 1 Department of Physiology, College of Medicine, Hallym University, Chuncheon 24252, Korea; jd1422@hanmail.net (J.H.J.); bychoi@hallym.ac.kr (B.Y.C.); rnlduadkfk136@hallym.ac.kr (A.R.K.); sshlee@hallym.ac.kr (S.H.L.); zxnm01220@gmail.com (D.K.H.); bluesea3616@naver.com (S.H.L.); 2 Department of Medical Science, College of Medicine, Hallym University, Chuncheon 24252, Korea; 3 Faculty of Medical Sciences, Western University, London, ON N6A 5C1, Canada; sam8233157@gmail.com; 4 College of Medicine, Neurology, Hallym University, Chuncheon 24252, Korea; hksong0@paran.com (H.K.S.); dohchi@naver.com (H.C.C.)

**Keywords:** epilepsy, pilocarpine, donepezil, neuron death, oxidative injury, microglia activation

## Abstract

Epileptic seizures are short episodes of abnormal brain electrical activity. Many survivors of severe epilepsy display delayed neuronal death and permanent cognitive impairment. Donepezil is an acetylcholinesterase inhibitor and is an effective treatment agent for Alzheimer’s disease. However, the role of donepezil in seizure-induced hippocampal injury remains untested. Temporal lobe epilepsy (TLE) was induced by intraperitoneal injection of pilocarpine (25 mg/kg). Donepezil (2.5 mg/kg/day) was administered by gavage in three different settings: (1) pretreatment for three days before the seizure; (2) for one week immediately after the seizure; and (3) for three weeks from three weeks after the seizure. We found that donepezil showed mixed effects on seizure-induced brain injury, which were dependent on the treatment schedule. Pretreatment with donepezil aggravated neuronal death, oxidative injury, and microglia activation. Early treatment with donepezil for one week showed neither adverse nor beneficial effects; however, a treatment duration of three weeks starting three weeks after the seizure showed a significant reduction in neuronal death, oxidative injury, and microglia activation. In conclusion, donepezil has therapeutic effects when injected for three weeks after seizure activity subsides. Therefore, the present study suggests that the therapeutic use of donepezil for epilepsy patients requires a well-conceived strategy for administration.

## 1. Introduction

The most common form of non-congenital epilepsy, temporal lobe epilepsy (TLE), can be initiated by a variety of brain insults such as hypoxia, head trauma, or multiple febrile seizure [1]. TLE is one of the most debilitating common neurological illnesses experienced by young people across the world and is one of the principal causes of cognitive impairment [2]. Epilepsy consists of several distinct neurological disorders that all share forms of epileptic seizures [3,4,5]. The root cause of epilepsy remains unidentified, although many patients develop epilepsy after brain damage such as stroke and traumatic brain injury, which is possibly caused in part by an up-regulation of excitatory neurotransmission, or the down-regulation of inhibitory neurotransmission, subsequent to the above brain insults [6]. Continued and uncontrolled epilepsy can cause permanent brain damage and subsequent worsening of cognitive function [5]. In particular, epilepsy has a profound deteriorative effect on hippocampal function, which is susceptible to neural damage and gradually accumulates harmful cellular and metabolic changes [7,8]. Moreover, epilepsy has been shown to alter neural development and synaptic reconstruction in the hippocampus, leading to increased spontaneous seizures [9]. Survival of severe epilepsy has improved over the past few decades through modern clinical approaches including anticonvulsant drugs and antibiotic treatment [10]. Nevertheless, many survivors who have experienced severe epilepsy still show neuronal injury and cognitive impairment.

An acetylcholinesterase inhibitor, donepezil, is a drug that inhibits the acetylcholinesterase enzyme from degrading acetylcholine, thereby increasing the action magnitude and time period of the neurotransmitter acetylcholine [11,12]. Donepezil, also called Aricept, has been shown to be an effective treatment agent for Alzheimer’s disease [13,14,15]; donepezil also affects the Alzheimer patient’s cognitive and behavioral function [16]. However, in patients with moderate-to-severe Alzheimer’s disease, continued treatment with donepezil showed minimal benefit [17]; therefore, the efficacy of this acetylcholinesterase inhibitor in the treatment of Alzheimer’s disease remains controversial [18]. Donepezil reversibly inactivates cholinesterase, thus inhibiting the hydrolysis of acetylcholine. Therefore, it increases the concentration of acetylcholine in the extrasynaptic space of cholinergic neurons. Donepezil also prevents neuronal death after traumatic brain injury [19]; however, the role of donepezil in seizure-induced neuronal death has not been clarified.

Our previous study showed that acetylcholine precursors such as citicoline and α–GPC demonstrated mixed effects on seizure behavior and seizure-induced neuronal death [20,21,22]. Citicoline and α–GPC both increased seizure-induced neuronal death if injected immediately after the seizure. However, if these acetylcholine precursors were injected when seizure activity had diminished, seizure-induced neuronal death decreased. Therefore, we proposed that the application of choline precursors in epilepsy patients needed special attention regarding timing. However, the effects of acetylcholinesterase inhibitors on seizure-induced neuronal death have yet to be tested. Thus, the aim of this study was to evaluate the effects of donepezil on brain injury after pilocarpine-induced seizure.

## 2. Results

### 2.1. Pre-Treatment of Donepezil for Three Days before Seizure Increases Neuronal Death, Oxidative Injury, and Microglia Activation

**Figure 1:**
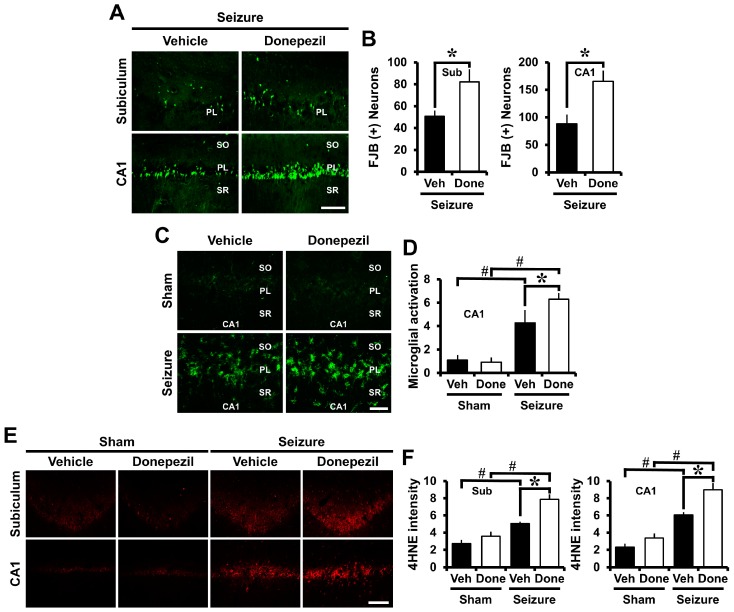
Pre-treatment of donepezil increased seizure-induced neuronal death, microglia activation and oxidative injury. (**A**) Representative images show the fluoro-jade B (FJB) (+) neurons in the CA1 and subiculum (Sub) of the hippocampus. The number of degenerating neurons was increased in the donepezil-treated group when compared to the vehicle-treated group. Scale bar = 100 μm; (**B**) The bar graph shows the number of degenerating neurons; (**C**) Fluorescent images indicate microglia activation in the CA1 of the hippocampus. In the group treated with donepezil after seizure, microglia activation was increased when compared to seizure vehicle group. Scale bar = 50 μm; (**D**) The bar graph was obtained by scoring the activation of microglia; (**E**) Fluorescent images indicate the 4HNE intensity in the CA1 and subiculum of the hippocampus. Oxidative injury increased in the donepezil group when compared to the vehicle group. Scale bar = 100 μm; (**F**) The bar graph shows the quantified 4HNE intensity. Data are mean ± S.E.M., *n =* 6 from each sham group. *n =* 5–7 from each seizure group. * Significantly different from vehicle-treated group. *^,#^ *p* < 0.05. SO (Stratum Oriense), PL (Pyramidal Layer), SR (Stratum Radiatum).

After severe seizures, neuronal death increases in the hippocampus. Fluoro-jade B (FJB) staining was performed to determine the pre-treatment effect of donepezil on seizure-induced neuronal death. Rats were sacrificed three days after pilocarpine-induced seizures. FJB staining is known as a marker for selectively detecting degenerating neurons [23]. Neuronal death was increased in the donepezil-treated group when compared to the vehicle group in the CA1 and subiculum (Sub) areas of the hippocampus (Figure 1A). As shown in Figure 1B, the number of degenerating neurons increased in the group receiving donepezil.

CD11b staining was performed to determine the pre-treatment effect of donepezil on seizure-induced microglia activation. CD11b staining is a form of immunofluorescent staining to confirm microglial activation. When the vehicle group and the donepezil group were compared after seizure, microglia activation was increased in the donepezil-treated group, more so than in the vehicle group (Figure 1C). As shown in Figure 1D, microglia activity—scored as per our criteria [24,25]—increased when the donepezil-treated group was compared to the vehicle group.

4-hydroxynonenal (4HNE) staining was performed to identify oxidative injury after seizure. 4HNE is a marker that selectively detects oxidative injury. The obtained tissues were stained using the 4HNE staining method to confirm oxidative injury in the CA1 and subiculum regions of the hippocampus. The group treated with donepezil showed higher oxidative injury than the seizure vehicle group (Figure 1E). As shown in Figure 1F, the intensity of 4HNE was higher in the donepezil group than in the vehicle group. These results indicated that administration of donepezil for three days before induction of seizure increased degenerating neurons, oxidative injury, and microglia activation after seizure.

### 2.2. Post-Treatment of Donepezil for One Week Had No Effects on Seizure-Induced Neuronal Death, Oxidative Injury, and Microglia Activation

**Figure 2:**
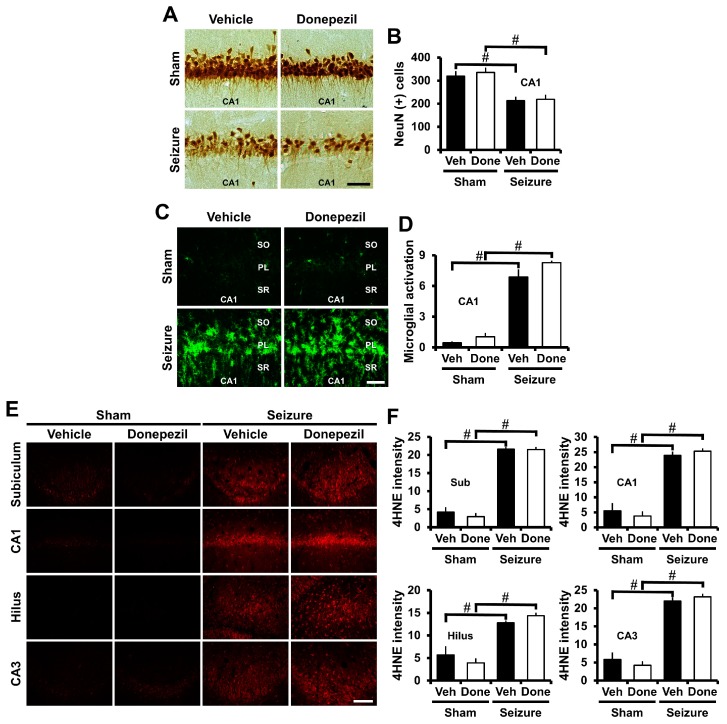
Post-treatment of donepezil for one week showed no effects on seizure-induced neuronal death, oxidative injury, and microglia activation. (**A**) Representative images indicate the NeuN (+) live neurons in the CA1 of the hippocampus. There was no difference in the number of live neurons between the vehicle-treated and donepezil-treated group after seizure. Scale bar = 50 μm; (**B**) The bar graph shows the number of NeuN (+) live neurons; (**C**) Fluorescent images indicate microglia activation in the CA1 of the hippocampus. There was no difference in microglia activation between the seizure vehicle and donepezil groups. Scale bar = 50 μm; (**D**) The bar graph was obtained by scoring the activation of microglia; (**E**) Fluorescent images indicate oxidative injury in the CA1, CA3, hilus, and subiculum of the hippocampus. There was no difference in the intensity of oxidative injury between the seizure vehicle group and donepezil group. Scale bar = 100 μm; (**F**) The bar graph shows the intensity of the oxidative injury. *n* = 5 from each sham group. *n* = 8–10 from each seizure group. ^#^ Significantly different from vehicle treated group. ^#^ *p* < 0.05.

NeuN staining was performed to observe live neurons to confirm that donepezil administration for one week affected neuronal death after the pilocarpine-induced seizure. NeuN staining is widely used as a method for specifically detecting live neurons [26,27,28]. One week after seizure induction, the rats were sacrificed and the number of live neurons quantified. The pilocarpine-induced seizure resulted in neuronal damage in the CA1 area of hippocampus. When comparing the number of live neurons in the seizure-vehicle and seizure-donepezil groups, we found no difference between the two groups (Figure 2A,B).

CD11b staining was performed to determine the post-treatment effect of donepezil for one week on seizure-induced microglia activation. After seizure, microglia activation occurred in the CA1 region of the hippocampus. Compared to sham groups, microglia activity was increased in both the donepezil-treated and the vehicle-treated groups. We did not find any difference between the two groups concerning seizure-induced microglial activation (Figure 2C,D).

4HNE staining was performed to determine if the administration of donepezil for one week after seizure had an effect on oxidative injury. Pilocarpine-induced seizures led to oxidative injury in the CA1, CA3, hilus, and subiculum of the hippocampus at one week after insult. There was no difference between the two groups when comparing oxidative injury between the seizure-vehicle group and the seizure-donepezil group (Figure 2E,F). Therefore, these results suggested that the administration of donepezil for one week after seizure did not alter neuronal death, oxidative injury, and microglia activation.

### 2.3. Post-Treatment of Donepezil for Three Weeks from Three Weeks after Seizure Reduced Neuronal Death, Oxidative Injury and Microglia Activation

**Figure 3:**
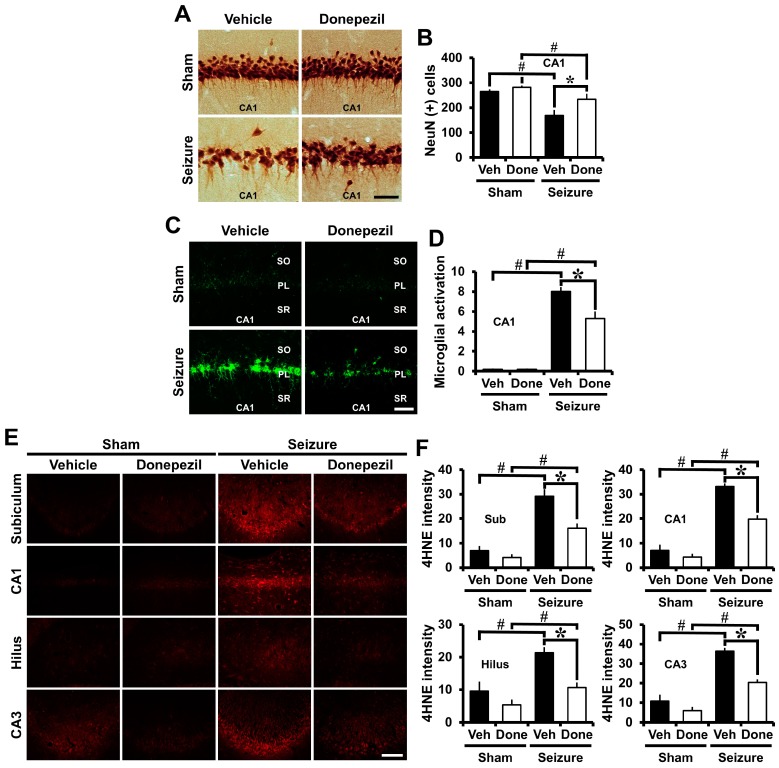
Post-treatment of donepezil for three weeks from three weeks after seizure reduced neuronal death, oxidative injury and microglia activation. (**A**) Representative images indicate the NeuN (+) live neurons in the CA1 of the hippocampus. Compared with the seizure-vehicle group, the number of live neurons increased after long-term administration of donepezil after seizure. Scale bar = 50 μm; (**B**) The bar graph shows the number of NeuN (+) live neurons; (**C**) Fluorescent images indicate microglia activation in the CA1 of the hippocampus. When compared with the seizure-vehicle group, microglia activation decreased with the long-term administration of donepezil after seizure. Scale bar = 50 μm; (**D**) The bar graph shows the quantification of the microglia activation; (**E**) Fluorescent images indicate oxidative injury in the CA1, CA3, hilus, and subiculum of the hippocampus. Compared with the seizure-vehicle group, the severity of oxidative injury was reduced by the long-term administration of donepezil after seizure. Scale bar = 100 μm; (**F**) The bar graph shows the intensity of the oxidative injury. Data are mean ± S.E.M., *n* = 5 from each sham group. *n* = 5–7 from each seizure group. * Significantly different from vehicle-treated group. *^,#^ *p* < 0.05.

NeuN staining was performed to confirm the neuroprotective effects of long-term administration of donepezil after seizure. Three weeks after seizure induction, donepezil was administered for three weeks and sacrificed at six weeks after seizure induction. When compared with the seizure-vehicle group, the number of live neurons was significantly increased in the CA1 region of the seizure-donepezil group (Figure 3A,B).

CD11b staining was performed to determine whether long-term administration of donepezil affected microglia activation after seizure. Brain tissue was obtained six weeks after pilocarpine-induced seizure, and immunofluorescent staining was performed to detect microglia activation. When compared to the seizure-vehicle group, microglia activation decreased in the donepezil-treated group (Figure 3C). As shown in the quantified graphs, donepezil treatment showed a significant reduction in microglia activation (Figure 3D).

4HNE staining was performed to determine whether long-term administration of donepezil affected oxidative injury after seizure. Oxidative injury was identified in the CA1, CA3, hilus, and subiculum of the hippocampus. When compared with the seizure vehicle group, the group treated with donepezil decreased oxidative injury (Figure 3E). Figure 3F shows that the donepezil-treated group showed a significant decrease in oxidative injury in each region of the hippocampus after seizure when compared to the vehicle-treated group. Therefore, prolonged administration of donepezil reduced neuronal death, oxidative injury, and microglia activation when initiated at three weeks post seizure.

## 3. Discussion

It is a well-established fact that persistent seizures induced by pilocarpine are followed by extensive damage to the whole brain [29]. It has been previously reported that donepezil, an acetylcholinesterase inhibitor, alleviates neuronal death in animal models such as ischemia [30] and traumatic brain injury [31], which cause severe damage to the brain, including seizures. However, the effect of donepezil on pilocarpine-induced seizure models has not been investigated. We investigated the effect of donepezil on pilocarpine seizure-induced neuronal death, oxidative injury, and microglia activation.

To confirm the effect of donepezil, the present study was divided into three settings: (1) pre-treatment (for three days before seizure); (2) short-term administration (for one week immediately after seizure); and (3) long-term administration (for three weeks from three weeks after seizure).

First, we administered donepezil for three days before inducing seizure to determine if there was a preventive effect against seizure-induced brain damage. Donepezil was orally administered at a concentration of 2.5 mg/kg for three days before seizure induction. We performed FJB staining, 4HNE staining, and CD11b staining to evaluate neuronal cell death after seizure. Each staining proceeded to confirm neuronal degeneration, oxidative injury, and microglia activation, respectively. As a result, treatment with donepezil for three days before inducing seizure with pilocarpine significantly increased neuronal death, oxidative injury, and microglia activation in the hippocampus. Thus, administration of donepezil before seizure induction caused adverse effects on neuronal death after seizure. Recently, it has been reported that donepezil acts to reduce neuronal death by decreasing p-CaMKII and p-CREB protein levels after ischemia [30]. There has also been a mechanistic report that donepezil is involved in the activation of nicotinic acetylcholine-receptors (nAChR), which affects the treatment of neuronal death and cognitive impairment after TBI [31]. However, the molecular mechanism involved in the adverse effects of donepezil on seizure-induced neuron death has yet to be fully understood. Pilocarpine-induced seizures are mediated by the M1 subtype of the muscarinic acetylcholine receptor (mAChR), and acetylcholine levels in the rat brain are increased during pilocarpine-induced seizures [32]. Acetylcholine acts as a neurotransmitter in the central nervous system and is involved in hippocampus mediated memory function [33,34]. Therefore, increased acetylcholine levels and activated cholinergic control by donepezil may exaggerate the deleterious effects of seizure given that donepezil is an acetylcholinesterase inhibitor, which increases the extrasynaptic levels of acetylcholine. When these two phenomena occur together in a short period of time, the level of acetylcholine in the brain increases in an abnormally rapid fashion.

Since all medications are not used as treatment before the onset of the disease, treatment with donepezil should be followed after the onset of seizure to evaluate the therapeutic effects of donepezil. Thus, we performed a short-term administration of donepezil for one week after seizure. Donepezil was orally administered for one week after seizure induction. Next, the number of live neurons, oxidative injury, and microglia activation were counted to determine the effect of donepezil on the neuronal death process in the hippocampus. In this study, we found that there was no difference between the two groups when comparing the seizure vehicle group and seizure donepezil group. Administration of donepezil for one week after seizure did not protect neuronal death, oxidative injury, and microglia activation. Thus, we concluded that short-term treatment of donepezil had neither beneficial effects nor adverse effects on neuronal death in pilocarpine-induced seizures. As the one-week period following seizure is the most active period of status epilepticus, donepezil did not protect against the neuronal death that occurs after seizure.

Acetylcholine levels were increased temporarily after seizure and then reduced at later time points. Therefore, we induced seizure with pilocarpine and treated donepezil for a long period (three weeks). After three weeks of seizure, donepezil was administered for three weeks. Repeated seizures were continued and maintained until three weeks after seizure. Thus, we administered donepezil treatment starting at three weeks after seizure. Since our previous study did not identify neuroprotective effects when an acetylcholine precursor was administered for three weeks immediately following seizure [21], we started donepezil injections from three weeks after the seizure event. Thus, in this study we administered donepezil for three weeks from three weeks after seizure induction to confirm the effects of long-term administration of donepezil. We quantified the number of live neurons, oxidative injury, and microglia activation to determine the long-term effects of donepezil. In this study, we found that if donepezil was given for three weeks after the seizure activity subsided, it reduced neuron degeneration, decreased oxidative injury, and decreased microglia activation.

We hypothesized that treatment with donepezil would reduce neuronal death in the hippocampus following pilocarpine-induced seizures. However, pre-treatment or treatment delivered immediately after the administration of donepezil while seizure activity was occurring did not show any beneficial effects on hippocampal damage after seizure, and the pretreatment of donepezil even aggravated hippocampal damage. In this study, we found that long-term administration of donepezil after seizure activity reduced the produced neuroprotective effects. Therefore, our study suggested that the therapeutic use of donepezil for epilepsy patients requires a careful injection schedule and consideration of how timing coincides with prevailing post-seizure acetylcholine levels.

## 4. Materials and Methods

### 4.1. Ethics Statement

This study was rigorously approved in accordance with the rules of the Laboratory Animals Guide and Laboratory Animals published by the National Institute of Health. Animal studies were conducted in accordance with the criteria from the Committee on Animal Habitation (Protocol # Hallym 2016–18). This study minimized the pain caused by urethane anesthesia when extracting the brain of animals and tried to minimize pain in all animal experimental areas.

### 4.2. Experimental Animals

This study used Sprague-Dawley male rats (250–300 g, DBL Co, Chungcheongbuk-do, Korea), aged 8 weeks. The animals were kept at room temperature (22 ± 2 °C) and constant humidity (55 ± 5%). Indoor lighting was set to automatically turn on at 12-h intervals (on at 8:00 and off at 20:00). This guideline was prepared in agreement with the ARRIVE (Animal Research: Reporting in Vivo Experiments) guidelines. We have previously described an effect of acetylcholine precursors on pilocarpine-induced seizure [21]. Based on that, we set up an experimental schedule as follows where animals were divided into four groups at each time point and the experiment was conducted as follows: (1) pre-treatment (sham-vehicle *n* = 6, sham-donepezil *n* = 6, seizure-vehicle *n* = 5, seizure-donepezil *n* = 7); (2) direct treatment after seizure (sham-vehicle *n* = 5, sham-donepezil *n* = 5, seizure-vehicle *n* = 8, seizure-donepezil *n* = 10); and (3) long-term treatment after seizure (sham-vehicle *n* = 5, sham-donepezil *n* = 5, seizure-vehicle *n* = 5, seizure-donepezil *n* = 7).

### 4.3. Seizure Induction

To examine the role of donepezil in neuronal death after pilocarpine-induced seizure, rats were intraperitoneally injected with lithium chloride (LiCl, Sigma-Aldrich Co., St. Louis, MO, USA, 127 mg/kg, i.p.) 19 hours before the injection of pilocarpine. Scopolamine (Sigma-Aldrich Co., St. Louis, MO, USA, 2 mg/kg, i.p.) was injected 30 min before pilocarpine injection, which was used to inhibit peripheral cholinergic properties. Pilocarpine (Sigma-Aldrich Co., St. Louis, MO, USA, 25 mg/kg i.p.) was intraperitoneally injected 30 min after scopolamine injection. Injection of pilocarpine induced status epilepticus (SE) [35]. SE classically occurred within 20–30 min of the pilocarpine injection [36]. The animals were placed at one animal per cage to observe seizure behavior. The seizure behavior was observed every 5 min according to the Racine process; Score 0 = no seizure behavior; Score 1 = stereotyped mouse and eye-blinking, facial exercise; Score 2 = head nodding; Score 3 = forelimb clonus; Score 4 = forelimb clonus and rearing; and Score 5 = rearing and falling [37]. Behavior changes from 0–5 were observed, and when the 5th step (falling) occurred, it was regarded as seizure onset. Diazepam (Valium, Hoffman-La Roche, Neuilly sur-Seine, France, 10 mg/kg, i.p.) was intraperitoneally injected 2 h after the start of SE. Some animals showed recurrent seizures after treatment with diazepam [38]. Despite the administration of diazepam, severe episodes of recurrent seizures were stopped by administering more diazepam (2 mg/kg, i.p.) [39].

### 4.4. Acetylcholinesterase Inhibitor (Donepezil) Treatment

Donepezil (2.5 mg/kg) was administered orally, which is the most commonly used method [28,40,41], to evaluate the effect of donepezil on neuronal death after pilocarpine-induced seizure. The concentrations of donepezil used have been previously described in other studies including Reference [42]. Experimental groups were divided into four groups: Sham-vehicle; Sham-donepezil; Seizure-vehicle; and Seizure-donepezil. The vehicle group was orally administered 0.9% saline instead of donepezil. To confirm the effect of donepezil, our study was divided into three settings: (1) pre-treatment (for three days before seizure); (2) short-term administration (for one week immediately after seizure); and (3) long-term administration (for three weeks from three weeks after seizure).

### 4.5. Brain Sample Preparation

Animals were sacrificed at three days, one week, and six weeks after seizure. Animals were anesthetized by injection of urethane (1.5 g/kg, i.p.). After anesthesia, animals were transcardially perfused with 600 mL saline, and then with 600 mL of 4% paraformaldehyde. The brains were quickly removed and fixed in the same 4% paraformaldehyde for 1 h. After fixation, a 30% sucrose solution, which acts as a cryoprotectant, was added to the solution. Two days later when the brain was submerged on the floor, the entire brain was frozen by dry ice. The whole brain was cut in the cryostat at a thickness of 30 μm and stored in a stock solution until the histological evaluation was in progress.

### 4.6. Detection of Neuronal Death

FJB staining was performed to decide the effect of donepezil on neuronal death after pilocarpine-induced seizure. FJB staining was carried out as defined by Schmued et al. [43,44]. Brain tissues were obtained at three days after the seizure. The obtained tissues were put on a slide coated with gelatin and dried. The slide was then dipped in alcohol and soaked in a 0.06% potassium solution for 15 min. Next, samples were immersed in a 0.001% FJB (Histo-Chem Inc., Jefferson, AR, USA) solution for 30 min, dyed, and washed with distilled water. The stained tissue was examined with a blue light microscope with a light wavelength of 450–490 nm using an Axioscope microscope (Carl Zeiss, Munchen Hallbergmoos, Germany). Quantification of FJB positive neurons was conducted by a blind observer. Degenerating neurons were quantitated by counting the number of FJB (+) neurons in the hippocampal CA1 and subiculum regions.

### 4.7. Detection of Oxidative Injury

To detect oxidative injury, 4HNE staining was performed at three days, one week, and six weeks after pilocarpine-induced seizures. Oxidative injury was assessed by measuring the intensity of 4HNE staining. The brain tissues were cut to a thickness of 30 μm using a cryostat, then stained. The obtained tissues were rinsed with phosphate buffered saline (PBS) and pretreated with a solution containing 90% methanol, distilled water, and 30% hydrogen peroxide to completely remove blood from the blood vessels in the tissues. Immunohistochemical staining with the 4HNE (diluted 1:500; Alpha Diagnostic Intl. Inc., San Antonio, TX, USA) antibody was performed as described in previous studies in References [45,46]. Brain tissue was immersed in a solution of monoclonal mouse anti-4HNE antiserum in PBS containing 0.3% Triton X-100 and kept overnight at 4 °C. Tissues were rinsed three times for 10 min with PBS, and the tissue was diluted 1:250 with Alexa Fluor 594 donkey anti-mouse immunoglobulin G (IgG) secondary antibody (Invitrogen, Grand Island, NY, USA) in PBS containing 0.3% Triton X-100 and treated for 2 h. The stained tissue was inspected on a gelatin-coated slide. Quantification of the stained tissue was undertaken using the Image J (v1.6.0) program for detecting the intensity of 4HNE.

### 4.8. Detection of Microglia Activation

CD11b staining was carried out to determine whether donepezil administration had any effect on microglia activation. Brain tissues were obtained at three days, one week, and six weeks after the seizure. The obtained tissues were rinsed with phosphate buffered saline (PBS) and pretreated with a solution containing 90% methanol, distilled water, and 30% hydrogen peroxide to completely remove blood from the blood vessels in the tissues. The tissues were immersed in a solution of monoclonal mouse anti-rat CD11b antiserum (diluted 1:500; AbD Serotec, Oxford, UK) in PBS containing 0.3% Triton X-100 and kept overnight at 4 °C. Tissues were rinsed three times for 10 min with PBS, and then immersed in a solution containing 1:250 with Alexa Fluor 488-conjugated donkey anti-mouse IgG secondary antibody (Invitrogen, Grand Island, NY, USA) with 0.3% Triton X-100 for 2 h. Microglial activation was measured by a blind observer. Each sample was made up of five sections on a slide, and each evaluation was graded on five tissues. Microglial activation was scored based upon the number of CD11b immunoreactive cells, the intensity of fluorescence, and its morphology. Each of these criteria was scored from 0–3. The total score was the sum of the scores for each criterion and was expressed as a score from 0–9 [24,25].

### 4.9. Detection of Live Neurons

NeuN staining was performed to evaluate the neuroprotective effect of donepezil for one week or six weeks after pilocarpine-induced seizure. Brains were transcardially perfused at one week or six weeks after seizure. The cryostat sections were stored and brain tissues were washed in PBS solution three times for 10 min. The washed tissues were pretreated with a solution containing 90% methanol, distilled water, and 30% hydrogen peroxide. The brain tissues were immersed in a solution of monoclonal mouse anti-NeuN antiserum (diluted 1:500; Millipore Co., Billerica, MA, USA) in PBS containing 0.3% Triton X-100 and kept overnight at 4 °C. After rinsing the tissues three times for 10 min with PBS, the tissues were immersed in a solution of anti-mouse IgG (diluted 1:250; Burlingame, Vector, CA, USA) in PBS containing 0.3% Triton X-100 and treated for 2 h. Next, the ABC complex solution (Burlingame, Vector, CA, USA) was treated at room temperature for 2 h. It was confirmed that 3,3′-diaminobenzidine (DAB ager, Sigma-Aldrich Co., St. Louis, MO, USA) dissolved in 0.01 M PBS buffer was used for coloring for one minute and 30 s. The stained brain tissues were placed on a gelatin-coated slide and dried and mounted with Canada balsam. Immunoreactive stained tissues were observed using an Axioscope microscope (Carl Zeiss, Munchen Hallbergmoos, Germany). Live neurons were identified by a blind observer in the CA1 areas of the hippocampus.

### 4.10. Data Analysis

Statistical significance between the experimental groups was determined by variance (ANOVA) according to the Bonferroni post hoc test (IBM SPSS Statistics software, Armonk, NY, USA). Data were expressed as mean ± S.E.M., and the differences were considered significant at *p* < 0.05.
